# Investigation of phase composition and nanoscale microstructure of high-energy ball-milled MgCu sample

**DOI:** 10.1186/1556-276X-7-390

**Published:** 2012-07-13

**Authors:** Zongqing Ma, Yongchang Liu, Liming Yu, Qi Cai

**Affiliations:** 1 Tianjin Key Laboratory of Composite and Functional Materials, School of Materials Science and Engineering, Tianjin University, Tianjin, 300072, People’s Republic of China

## Abstract

The ball milling technique has been successfully applied to the synthesis of various materials such as equilibrium intermetallic phases, amorphous compounds, nanocrystalline materials, or metastable crystalline phases. However, how the phase composition and nanoscale microstructure evolute during ball milling in various materials is still controversial due to the complex mechanism of ball milling, especially in the field of solid-state amorphization caused by ball milling. In the present work, the phase evolution during the high-energy ball milling process of the Mg and Cu (atomic ratio is 1:1) mixed powder was investigated. It was found that Mg firstly reacts with Cu, forming the Mg_2_Cu alloy in the primary stage of ball milling. As the milling time increases, the diffracted peaks of Mg_2_Cu and Cu gradually disappear, and only a broad halo peak can be observed in the X-ray diffraction pattern of the final 18-h milled sample. As for this halo peak, lots of previous studies suggested that it originated from the amorphous phase formed during the ball milling. Here, a different opinion that this halo peak results from the very small size of crystals is proposed: As the ball milling time increases, the sizes of Mg_2_Cu and Cu crystals become smaller and smaller, so the diffracted peaks of Mg_2_Cu and Cu become broader and broader and result in their overlap between 39° and 45°, at last forming the amorphous-like halo peak. In order to determine the origin of this halo peak, microstructure observation and annealing experiment on the milled sample were carried out. In the transmission electron microscopy dark-field image of the milled sample, lots of very small nanocrystals (below 20 nm) identified as Mg_2_Cu and Cu were found. Moreover, in the differential scanning calorimetry curve of the milled sample during the annealing process, no obvious exothermic peak corresponding to the crystallization of amorphous phase is observed. All the above results confirm that the broad halo diffracted peak in the milled MgCu sample is attributed to the overlap of the broadened peaks of the very small Mg_2_Cu and Cu nanocrystalline phase, not the MgCu amorphous phase. The whole milling process of MgCu can be described as follows: $\text{Mg}+\text{Cu}\to {\text{Mg}}_{2}\text{Cu}+\text{Cu}\to {\text{Mg}}_{2}{\text{Cu}}_{\text{nanocrystal}}+{\text{Cu}}_{\text{nanocrystal}}$.

## Background

The mechanical alloying (MA) process developed by Benjamin et al. [1,2] in the early 1970s is now recognized as a versatile technique for obtaining oxide dispersion-strengthened superalloys, equilibrium intermetallic phases, amorphous compounds, nanocrystalline materials, or metastable crystalline phases. Due to the complicated ball milling environment, how the nanoscale microstructure evolutes during the ball milling in various materials is still under discussion, and some conclusions on the final phases after ball milling are controversial, especially in the field of solid-state amorphization caused by ball milling [3,4].

On the other hand, in the past two decades, Mg-based amorphous alloys (Mg-Cu-Y, Mg-Ni, Mg-Cu, etc.) are regarded as a new family of promising materials with excellent specific strength, improved hydrogen storage, and good corrosion resistance [5]. Considering the large differences in melting points and vapor pressures between Mg and other alloying elements, it is a great challenge to obtain Mg-based amorphous alloys by traditional casting techniques. MA is a low-temperature process; therefore, it overcomes the disadvantages of conventional alloying and allows forming amorphous samples for compositions which cannot be amorphized by casting techniques. In fact, a number of binary or ternary Mg-based amorphous alloys, such as Mg-Ni [6] and Mg-Cu-Y [7,8], have been synthesized by mechanical alloying of the crystalline elemental powders. Some previous studies [9,10] reported that the MgCu amorphous alloys could also be prepared by ball milling. They considered the final product of a milled MgCu sample as amorphous alloy based on the broad halo peak in the X-ray diffraction pattern alone. However, it should be noted that it is not possible to distinguish among the materials which are (a) truly amorphous and (b) extremely refined grain by observing the broad X-ray peaks alone [3], especially in the ball-milled samples. Hence, the above conclusions on the ball-milled MgCu sample might not be very valid.

Based on above background, from the perspective of the development of Mg-Cu amorphous alloys, and also on the understanding of solid-state amorphization mechanism during ball milling process, it makes sense to clarify the phase composition and nanoscale microstructure of the high-energy ball milled MgCu. Hence, in the present work, the phase evolution during the high-energy ball milling process of the Mg and Cu mixed powder was investigated. Furthermore, microstructure observation and annealing treatment of the milled MgCu sample were also carried out.

## Methods

The Mg powder (99.8cs% purity, 325 mesh) and Cu powder (99.9% purity, 625 mesh) were mixed in a molar ratio of Mg_50_Cu_50_. Then, tungsten carbide milling balls and the mixed powder were put into the tungsten carbide vessel with the ball-to-powder weight ratio of 5:1 in the argon box. The high-energy ball milling was performed on a SPEX 8000 M mill (Thomas Scientific, Swedesboro, NJ, USA) under argon atmosphere. The milling process was performed in a discontinuous way consisting of 1 milling h followed by rest period of 0.5 h. The powders after milling for several different times were characterized by X-ray diffraction (XRD) in the Bruker D8 Advance X-ray diffractometer (Bruker Optik GmbH, Ettlingen, Germany). The evolution of grain size of Mg and Cu during the ball milling was estimated using the single-line method of diffraction line-broadening analysis based on the XRD data.

The microstructure was investigated using high-resolution transmission electron microscopy. The differential scanning calorimetry (DSC) measurements of the milled MgCu powders were carried out using the power-compensated PerkinElmer Pyris-1 (PerkinElmer, Waltham, MA, USA) from 50°C to 450°C with the heating rate of 20°C/min under argon gas protection. Accordingly, several annealing temperatures were determined, and the annealing experiment is carried out at these temperatures. After that, the phase composition of the annealed samples is also studied by X-ray diffraction.

## Results and discussion

**Figure 1:**
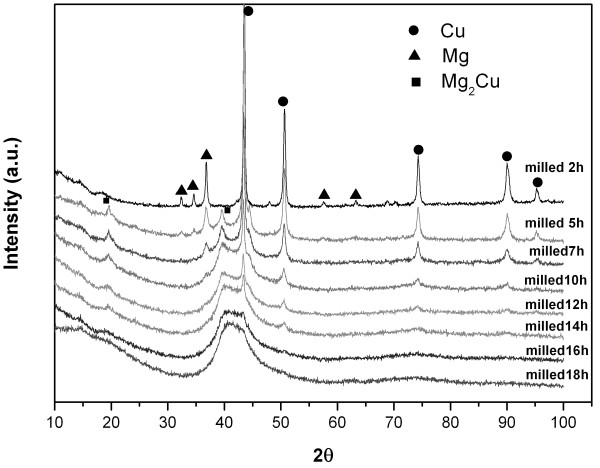
XRD patterns of MgCu sample after ball milling for different times.

**Figure 2:**
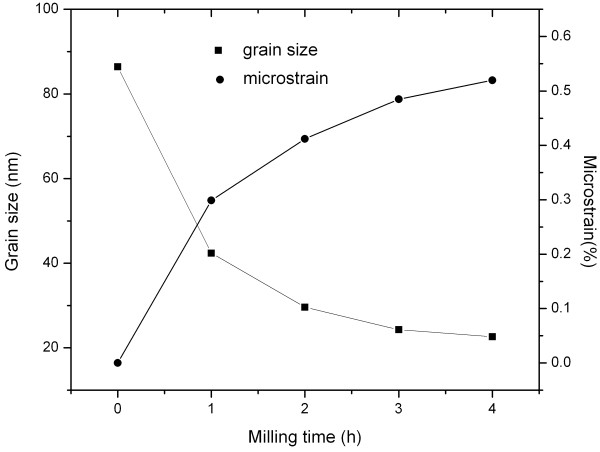
Evolution of grain size and microstrain in Mg during ball milling of the MgCu sample.

**Figure 3:**
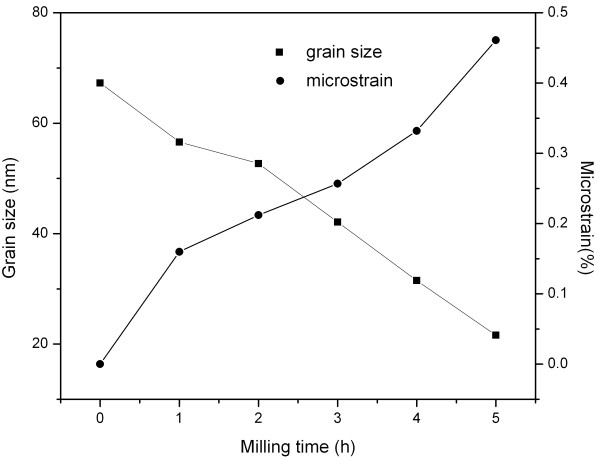
Evolution of grain size and microstrain in Cu during ball milling of the MgCu sample.

Figure 1 shows the XRD patterns of MgCu samples after ball milling for different times. It is found that some of Mg firstly reacts with Cu, forming the Mg_2_Cu alloy in the primary stage of ball milling. As the milling time increases, the intensities of Mg, Mg_2_Cu, and Cu peaks gradually decrease, and the corresponding widths broaden, which is mainly attributed to the grain refinement and accumulation of microstrain during the ball milling. The evolution of grain size and microstrain in the Mg and Cu is estimated using the single-line method of diffraction line-broadening analysis and illustrated in Figures 2 and 3, respectively (this estimation was only applied to the short-time milled samples for the reason that the diffracted peaks of Mg and Cu become unobvious when the ball milling time is longer than 5 h). One can see that the grain sizes of Mg and Cu both decrease, while their microstrains increase significantly as the ball milling proceeds. When ball milling time reaches 18 h, all the peaks of Mg, Mg_2_Cu, and Cu cannot be recognized, and a broad halo peak appears in the XRD pattern. In previous studies [9,10], it was suggested that the halo peak originated from the amorphous phase formed during the ball milling. However, considering the asymmetric shape of this broad halo characteristic of amorphous materials, another possibility that this halo peak results from the very small size of Mg_2_Cu and Cu crystals is proposed in the present work: As the ball milling time increases, the sizes of Mg_2_Cu and Cu become smaller, and consequently, the diffracted peaks of Mg_2_Cu and Cu between 39° and 45° become so broad that they could overlap each other, finally forming the amorphous-like halo peak. Other peaks of Mg_2_Cu and Cu cannot be found mainly due to the small size of Mg_2_Cu and Cu nanocrystals (as discussed above, the size of grains have already decreased below 20 nm) after ball milling.

Based on the above discussion, what is the real origin of the broadened halo peak in the milled sample? Are nanocrystals or amorphous phase present in the ball-milled MgCu sample? To answer these questions, it is needed to further investigate the ball-milled sample with the aid of microstructure observation and annealing experiment.

**Figure 4:**
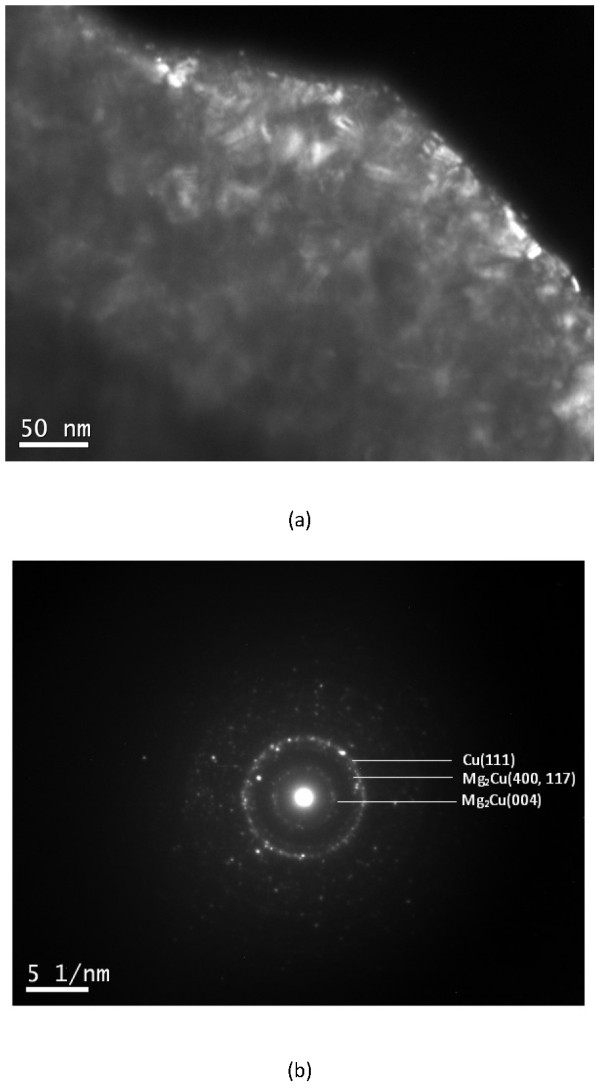
TEM dark-field image (a) and corresponding SAD pattern (b) of one 18-h milled MgCu particle.

**Figure 5:**
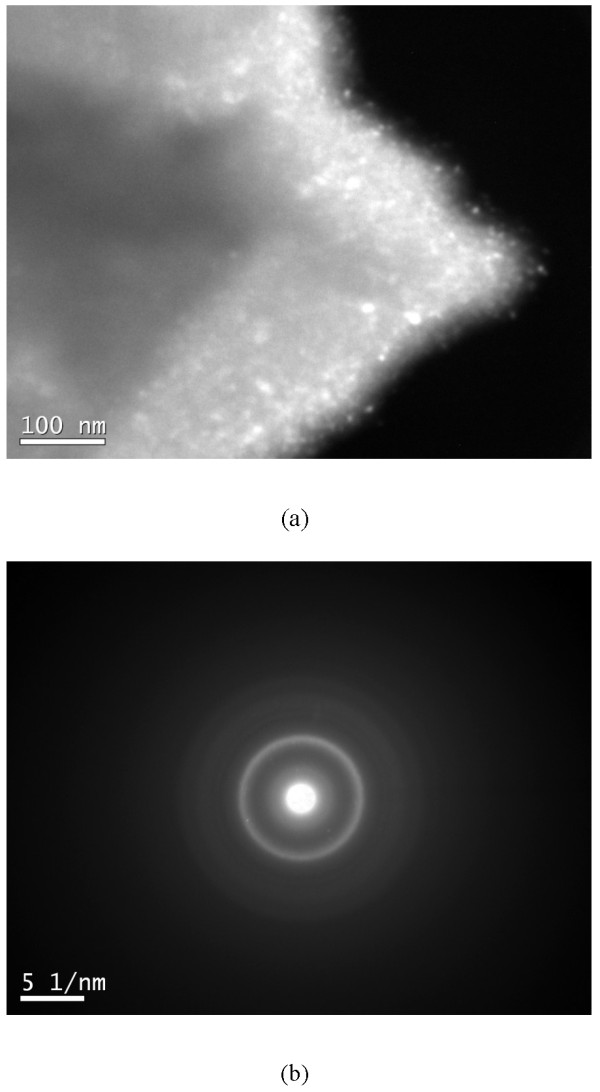
TEM dark-field image (a) and corresponding SAD pattern (b) of another 18-h milled MgCu particle.

Figures 4 and 5 show some representative transmission electron microscopy (TEM) images of different particles in the 18-h milled MgCu sample. According to the dark-field image and the corresponding selected area electron diffraction (SAD) pattern in Figure 4, the particle consisted of nanocrystals with a size of less 20 nm. Based on the SAD pattern, the nanocrystals are identified as Cu and Mg_2_Cu. The result is consistent with the XRD patterns in Figure 1, in which the peaks of Cu and Mg_2_Cu phase both gradually become broader and finally unrecognized due to the formation of Cu and Mg_2_Cu nanocrystals.

The microstructure of the particle in Figure 5 seems different from the particle in Figure 4. The rings in the SAD pattern (see Figure 5b) are more diffused, and no clear ring can be observed. Only a halo is present in the SAD pattern. This type of pattern is always identified as amorphous in the literature. However, in the corresponding dark-field image of this particle (see Figure 5a), the presence of bright dots indicates that many nanocrystals still exist in this particle (about 10 nm). Observing Figure 5b more carefully, it can be found that the halo is located in almost the same position of the rings belonging to Cu and Mg_2_Cu (see Figure 4b). Hence, it is speculated that the diffraction of a large amount of very small Cu and Mg_2_Cu nanocrystals possibly results in the amorphous-like pattern in the Figure 5b. Different microstructures in the Figures 4 and 5 from the same milled sample also imply that the size distribution of grains after ball milling is not uniform.

**Figure 6:**
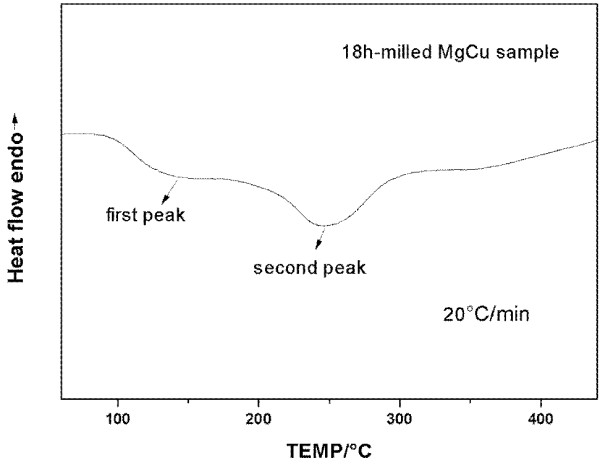
**Measured DSC curves of 18-h milled MgCu sample.** The sample was heated from 50°C to 450°C with a heating rate of 20°C/min.

**Figure 7:**
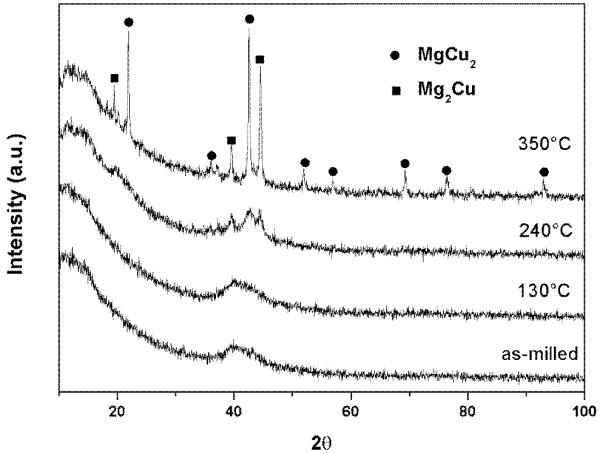
XRD pattern of 18-h milled MgCu sample annealed at different temperatures.

The DSC curve of the 18-h milled MgCu sample during the heating process is present in Figure 6. There are two exothermal peaks appearing in the DSC curve. In order to determine the origin of these two exothermal peaks, annealing experiments on 18-h milled MgCu samples were carried out at different temperatures, and the phase composition of annealed samples was studied by X-ray diffraction. One can see that no obvious change can be found in the XRD patterns of the as-milled sample and sample annealed at 130°C (the ending point of the first exothermal peak) except that a minor peak at about 40° appears (on the halo peak) in the XRD pattern of the sample annealed at 130°C (see Figure 7). This peak was identified as the main peak of the Mg_2_Cu phase. It is explained that as annealing is performed, Mg_2_Cu nanocrystals start to grow, and they become so large that they can be detected by X-ray diffraction. Hence, the first exothermal peak seems to be associated with the growth of the nanocrystals and also the relaxation of stress and should not result from the crystallization of the amorphous phase. On the other hand, one can see that the diffracted peaks of MgCu_2_ appear in the XRD pattern of the milled MgCu sample after annealing at 240°C (the peak point of the second exothermal peak). Moreover, as annealing temperature increases, the peak intensities of MgCu_2_ and Mg_2_Cu become stronger, and finally, they are the main crystalline phases in the sample annealed at 350°C (the ending point of the second exothermal peak). Combined with the above results, the second exothermal peak is related to the reaction between nanocrystalline Mg_2_Cu and Cu, forming MgCu_2_. There is no obvious exothermic peak corresponding to the crystallization of the amorphous phase in the whole DSC curve of milled sample.

## Conclusions

Based on the above analysis, it is concluded that the long-time milled MgCu sample consisted of Cu and Mg_2_Cu nanocrystals, and the halo peak in the XRD pattern of the 18-h milled sample ought to be attributed to the overlap of the broadened peaks of the Cu and Mg_2_Cu nanocrytals. The whole milling process of the MgCu system can be described as follows: $\text{Mg}+\text{Cu}\to {\text{Mg}}_{2}\text{Cu}+\text{Cu}\to {\text{Mg}}_{2}{\text{Cu}}_{\text{nanocrystal}}+{\text{Cu}}_{\text{nanocrystal}}$. According to the present research, it is worth noting that for the preparation of amorphous alloys from different kinds of metal using ball milling, it is not precise to consider the diffused halo peak appearing in the XRD pattern of milled samples as amorphous phase without the careful investigation of the microstructure. Even during the observation of the microstructure, the appearance of the diffused ring in the SAD cannot guarantee that the sample consisted of the amorphous phase. The diffraction of a large amount of nanocrystals with a very small size might also result in the amorphous-like SAD pattern.

## Competing interests

The authors declare that they have no competing interests.

## Authors’ contributions

ZQM and YCL contributed equally to this work. They designed most of this project and analyzed all the experimental data. LMY carried out the ball milling experiment and annealing experiment. QC is in charge of the analysis of phase composition and observation of microstructure. All authors read and approved the final manuscript.
