# Correction: Investigation of phase composition and nanoscale microstructure of high-energy ball-milled MgCu sample

**DOI:** 10.1186/1556-276X-8-186

**Published:** 2013-04-23

**Authors:** Zongqing Ma, Yongchang Liu, Liming Yu, Qi Cai

**Affiliations:** 1 Tianjin Key Laboratory of Composite and Functional Materials, School of Materials Science & Engineering, Tianjin University, Tianjin 300072, P R China

## Correction

In the Methods section of our published article [1], the evolution of grain size and microstrain in the Mg and Cu is estimated using the single-line method of diffraction line-broadening analysis. However, a very important reference is omitted, and this method founder’s publication should be cited here [2].

Moreover, the experimental results contained in this paper were obtained by the first author in cooperation with Dr. U. Welzel, Dr. E. Bischoff and Prof. Dr. E.J. Mittemeijer (all Max Planck Institute for Intelligent Systems) during the stay of the first author in the department of Prof. Dr. Mittemeijer. Thus the authors would like to express our gratitude to them in the Acknowledgements section of this published article [1].
